# Supplementary material for: Are carbon emissions trading and green financial instruments synergistic? -Comprehensive quantitative research based on content analysis
Source: PLoS One. 2024 Mar 7;19(3):e0298601. doi: 10.1371/journal.pone.0298601 (PMC10919601; doi:10.1371/journal.pone.0298601)
Supplement: S1 Data — (ZIP) [file pone.0298601.s001.zip › Data/地级市Z_F工作报告.zip!/╔╜╢1⁄2/╠⌐░▓╩╨/└┤╫╘╣1⁄2╓┌║┼í╛┬φ┐╦╩2╛▌═°í┐_2015.docx]

各位代表：

　　现在，我代表市人民政府向大会报告工作，请予审议，并请市政协委员和列席会议的同志提出意见。

　　一、2014年工作回顾

　　2014年，面对复杂严峻的形势，在市委坚强领导下，全市上下紧紧围绕推进富民强市、建设幸福泰安奋斗目标，攻坚克难，真抓实干，经济社会发展取得新成绩。

　　经济保持稳定增长，发展质量逐步提升。全市实现生产总值3002.2亿元，增长9.4%。三次产业结构调整为9.04：48.22：42.74，三产提高1.07个百分点。完成地方一般公共预算收入187.4亿元，增长11%，其中税收占比达到72.2%，提高2个百分点，主体税收比重达到53.2%。完成固定资产投资2299亿元，增长16%。实现社会消费品零售总额1188.1亿元，增长12.8%。城镇居民人均可支配收入、农村居民人均纯收入分别达到30715元、12913元，增长8.9%和11.8%。人民币各项存、贷款余额分别为2458.4亿元、1591.6亿元，比年初增长8.7％和12.4%。

　　农业基础更加稳固，现代农业加快发展。粮食总产达到281.7万吨，实现“十二连增”，岱岳区、肥城市、宁阳县、东平县被表彰为全国粮食生产先进单位。全市新增茶园7000亩、高档苗木花卉2.8万亩、奶牛2万头；新认证无公害农产品5.4万亩、绿色食品8.2万亩、有机食品1.2万亩；新增农产品地理标志11件。新增农民合作社1327家、家庭农场943家、规模以上农业龙头企业114家。澳亚牧场、益客食品、东方园林、滕头园林等一批龙头企业和泰安国家农业科技园区、肥城市现代农业示范区、天泽农业科技园、泰茶“良心谷”生态示范园等一批特色园区发展较好，带动作用明显。休闲农业加快发展，岱岳区、东平县被评为全国休闲农业与乡村旅游示范县。全市推广农业生产新技术10余项，引进、示范新品种50多个。完成各类水利工程7060 项，建设“旱能浇、涝能排”高标准农田25万亩，解决了15.2万农村人口饮水安全问题。新建改建农村公路907.5公里。农村集体“三资”管理得到加强，新泰市被评为全国农村集体“三资”管理示范县。新型职业农民培训、扶贫开发取得新成效。强农惠农富农政策全面落实。

　　工业转型成效明显，创新驱动深入实施。规模以上工业企业实现主营业务收入7115.1亿元、利税768.3亿元、利润464.4亿元，分别增长12.7%、6.9%和3.6%。新增主营业务收入过10亿元企业3家、利税过亿元企业26家。实现民营经济增加值1636.3亿元，增长10.5 %。装备制造业、电子信息产业主营业务收入分别增长19.5%和20.7%。高新技术产业产值占比提高1个百分点以上。国家新能源示范城市、泰安高新区分布式光伏发电应用示范区加快建设。我市被确定为全国资源综合利用“双百工程”示范基地。新泰市被确定为全国首批循环经济示范创建市。全面完成了省下达的淘汰落后产能和节能减排目标任务。

　　科技创新能力进一步增强。全市实施技术创新项目1100余项，研发投入占生产总值比重达到2.15%，技改投资增长17.3%。国家级高新技术企业达到103家，国家级企业技术中心达到4家，省级工程技术研究中心达到42家。康平纳筒子纱数字化自动染色成套技术与装备荣获国家科技进步一等奖。商标战略深入实施，新增中国驰名商标5件、省著名商标14件。泰安高新区突出高端项目引进，国家“千人计划”智能机器人和激光器制造等一批战略性新兴产业项目先后签约入区。

　　服务业发展势头强劲，带动作用逐步增强。服务业投资占固定资产投资比重达到53.5%，服务业税收占比达到46.1%。旅游业初步改善了“山热城冷、昼热夜冷、夏热冬冷”局面，“三日游”影响力持续增强，游客满意度指数保持全省前列。全市共接待游客5311.8万人次，实现旅游总收入500.5亿元，分别增长10%和13.3%，泰山游客量首次突破500万人次。现代商贸物流业加快发展，中国物流泰安国际物流园、泺亨（泰安）现代物流中心、速恒物流园等一批物流项目和园区正在建设，新矿物流中心进入全国物流企业百强；泰山钢网、三星物流、杞农云商、泰山义乌购等本土电商企业快速成长。

　　重点项目加快建设，招商引资力度加大。全市122个市级重点建设项目开工120个，完成投资428.6亿元；100个前期推进项目开工65个。泰安如意科技时尚产业园、泰山玻纤产业园、国电热电联产、奥特莱斯现代服务产业区、华新现代服务城等项目顺利建设。借助全国工商联十一届三次执委会议暨全国知名民营企业家助推山东转调创投资洽谈会，面向民营企业大力开展招商活动，引进8家全国工商联执委企业和114家省外知名民营企业。成功举办了中国泰安投资合作洽谈会、（深圳）泰安经济合作洽谈会、中国（泰山）国际矿山装备与技术展览会等重大活动，签约了一批新项目。全市新开工亿元以上境内招商引资项目350个，同比增加69个；新开工外商投资项目79个，同比增加22个。

　　重点改革积极推进，开放水平显著提高。实施了市、县政府职能转变、机构改革和行政审批制度改革，承接国务院和省政府下放行政审批事项94项，取消40项，下放20项。推进财税制度改革，完善政府预算体系，公开了市级和6个县市区本级财政预决算、“三公”经费预决算总额，以及67个市直部门和281个县直部门预决算、“三公”经费预决算；加强了财政审计监督，全市财政拨款安排的“三公”经费同比下降16.4%；积极开展“营改增”试点扩围，各项结构性减税政策得到落实。创新国资监管体制，完善了监管机构、投资运营公司和实体企业“三位一体”改革架构，市级国有资本经营预算收入增长71.8%。实施商事制度改革，各类市场主体新登记数量增长60.9%。全市81.1%的村庄完成农村土地承包经营权确权登记颁证，东平县被确定为第二批全国农村改革试验区。深化集体林权配套改革和国有林场改革，徂徕山林场被评为全国十佳国有林场。城乡居民基本医疗保险实现并轨。启动了第二批县级公立医院综合改革。

　　开放型经济实现新突破。全市合同利用外资7.8亿美元，实际到账4.1亿美元，分别增长49%和34.6%。实现进出口总额29.7亿美元，其中出口17.3亿美元、进口12.4亿美元，分别增长19.5%、26.7%和10.8%。完成境外投资1.6亿美元，增长53.4%。泰安高新区海关综合监管区、青岛保税港区泰安（新泰）功能区建设积极推进。

　　城乡统筹步伐加快，城乡面貌有新变化。全市城镇化率达到55%。泰城规划实施了104国道改建、徂徕山汶河景区开发，分别拓展发展空间100多平方公里，城市框架进一步拉开。坚持实施片区开发、组团建设，万达城市广场、天房美郡、财源街中央商务区、中国五矿、雨润国际广场5个大型城市综合体和高铁新区、新华片区、万官片区、碧霞国际社区等10个城市片区加快推进。泰山国际会展中心建成运营。万官大街、花园路、温泉路南段、双龙路等顺利建设。引水入城工程全线贯通。西区“汽改水”全面完成。奈河景观提升工程效果显现。龙潭路地下停车场、6处农贸市场建成使用。餐厨垃圾处理厂投入运行。泰城新增139辆公交车，实行了 “一小时免费·优惠换乘”。

　　大力发展县域经济，肥城市被确定为全国主体功能区工作试点县，新泰市被确定为全省县域经济科学发展试点县。积极推进小城镇发展，13个全国重点镇和10个省级示范镇建设加快。全市建成农村新型社区266个，完成农房集中建设4.7万户、危房改造1.17万户。加大城乡环境综合整治力度，深入推进生态文明乡村行动和生态文明示范区建设，69个乡镇建设了垃圾中转站，3386个村（居）纳入城乡环卫一体化；累计建成省级以上生态乡镇58个、省级生态村18个；新泰市、肥城市创建国家卫生城市通过验收。积极开展国家森林城市创建，完成成片造林23.5万亩，森林覆盖率达到39.1%，汶河、康王河两处湿地被批建为国家湿地公园。实施大气污染防治“蓝天工程”三年行动计划，空气质量在全省内陆城市保持领先。

　　民生得到持续改善，社会建设不断加强。全市民生支出达到168.6亿元，占公共财政支出的59%。为民要办的10件实事，除泰安一中新城校区正积极推进外，其他9件全面完成。全市新增城镇就业6.2万人、农村劳动力转移就业7.9万人。收缴社会保险费133.7亿元，发放社会保险金114.2亿元；居民养老保险基础养老金由每人每月65元提高到75元；城市月低保、农村年低保、农村五保供养标准分别提高50元、625元和1000元。全市新建老年人日间照料中心72处、农村幸福院506处，新增养老床位13230张，山东泰山社会福利中心建设顺利。开工保障性住房10514套，基本建成7700套。发放住房公积金贷款11.6亿元。

　　学前教育更加普惠，公办及公办性质幼儿园达到81.5%；义务教育更加均衡，肥城市、宁阳县、新泰市通过国家义务教育均衡发展评估验收；普通高中教育质量进一步提升；职业教育加快发展。国家基本药物制度全面实施，人均基本公共卫生服务标准提高到35元；疾病预防控制、卫生应急工作得到加强，新泰市、肥城市被确定为国家慢性病综合防控示范区；泰安市妇幼保健院主体完工，山东中西医结合大学落户泰安。“群众文化艺术年”活动深入开展，公共文化基础设施加快建设，农家书屋覆盖率达到100%，泰山区、肥城市被评为全国文化先进县，东平戴村坝入选世界文化遗产名录。稳妥实施单独二孩政策，积极开展全国创建幸福家庭试点，我市连续13次获得全省人口目标责任考核先进单位称号。群众体育和竞技体育水平有新提高，泰山国际登山节被评为中国体育旅游十佳精品赛事。慈善事业加快发展，我市首次荣登中国城市公益百强榜。高度重视民族团结进步，新泰市荣获全国民族团结进步模范集体称号。双拥共建、国防动员、人民防空、老龄、妇女儿童、助残、宗教管理、外事侨务等工作都取得新成绩。

　　创新社会组织管理，我市被确定为全国首批社会组织建设创新示范区，“菜单式”志愿服务经验在全国推广。建立了部门综合大救助机制，“平台式”综合救助工作走在全国前列。深入推进平安泰安建设，“天网工程”在全省率先完成，民生警务平台运行良好。县级矛盾排查调处中心和乡镇、村居调解组织不断完善，人民调解作用有效发挥。理顺食品药品监管体制，市县乡村四级监管体系基本建立。完善安全监管长效机制，加大安全隐患排查整治力度，连续3年杜绝了较大以上事故发生。

　　工作作风进一步转变，服务效能明显提高。扎实开展党的群众路线教育实践活动，严格落实中央八项规定，集中开展“庸懒散”专项整治，“四风”问题得到有效整改。大力整治文山会海，以市政府名义召开的会议同比减少36.6%。集中整治行政审批“五不”现象，规范中介机构，对建设项目实行扎口管理，项目审批时限进一步缩短。完善政府依法决策机制，持续抓好机关作风和政风行风评议、群众满意度调查、政务督查、应急管理、便民服务电话、工作日志等工作，政府依法履职能力和服务水平进一步提升。

　　各位代表，2014年是不平凡的一年，“三期叠加”和经济运行下行，对以资源型、重化工为主的我市经济，带来的影响更大，困难比预料的更多，但是结果比预想的要好，取得这样的成绩实属不易。这是市委科学决策、坚强领导的结果，是全市人民同心同德、团结奋斗的结果。在此，我代表市政府，向全市广大干部群众、各民主党派、工商联、无党派人士，向人民解放军和武警驻泰部队及驻泰单位，向关心支持泰安发展的港澳台同胞、海外侨胞及海内外朋友，表示崇高的敬意和衷心的感谢！

　　在总结成绩的同时，我们也清醒地看到面临的困难和问题：一是经济持续稳定增长的压力加大，尤其是工业生产经营困难，土地、资金、劳动力、资源环境等要素制约加剧，经济发展预期不容乐观。二是结构性矛盾仍然是制约经济较快发展的主要因素，产业结构偏重，产业链条短；产品附加值低，高科技产品少；企业创新能力不强，发展活力不足，转方式调结构任务仍很艰巨。三是体制性、机制性障碍依然存在，改革开放的力度需进一步加大。四是公共服务体系还不够完善，城市管理、社会治理、民生保障、安全生产等一些与群众切身利益相关的问题，解决得还不够好。另外，“四风”问题仍然存在，转变政府职能、改进工作作风、优化发展环境等还有许多工作要做。对这些问题，我们要认真研究解决。

　　二、2015年工作总体安排

　　今年是全面深化改革的关键之年，是全面推进依法治市的开局之年，也是全面完成“十二五”规划的收官之年，做好今年的政府工作意义重大。政府工作总要求是：全面贯彻落实党的十八大和十八届三中、四中全会以及习近平总书记系列重要讲话精神，按照中央、省经济工作会议和省“两会”要求，紧紧围绕推进富民强市、建设幸福泰安奋斗目标，坚持稳中求进工作总基调，以提高经济发展质量和效益为中心，以招商引资和项目建设为抓手，以增加财政收入和城乡居民收入为评价标准，主动适应经济发展新常态，坚定不移推动工作指导重大转变，强化创新驱动，加快转型升级；全面深化改革，扩大对外开放；加强民生保障，创新社会治理，努力保持经济持续健康发展和社会和谐稳定。

　　主要预期目标是：生产总值增长8.5%左右；地方一般公共预算收入增长9.5%左右；固定资产投资增长15%左右；社会消费品零售总额增长12.5%左右；进出口总额增长11%左右，实际利用外资增长18%左右；城镇居民人均可支配收入增长9%左右，农村居民人均纯收入增长10%左右；城镇新增就业5万人，城镇登记失业率控制在3.5%以内；人口自然增长率控制在10.39‰以内；全面完成省政府下达的节能减排任务。

　　当前，国内外经济形势依然错综复杂，世界经济仍处在金融危机后的深度调整期，复苏疲弱态势难有明显改观；我国经济发展进入从高速增长转向中高速增长的新常态，经济下行压力加大，结构调整阵痛显现，企业生产经营困难增多。但是，机遇与挑战并存。我国发展处于可以大有作为的重要战略机遇期没有变，经济发展总体向好的基本面没有变；中央全面推进改革，更大规模、更多方面的改革措施陆续落地，改革红利逐步释放；中央继续实施积极的财政政策和稳健的货币政策，这都为经济社会发展提供了有利环境和条件。新常态下，推动经济社会持续健康发展，一是必须更加注重消费、投资和出口的均衡协调拉动，发挥消费的基础性作用，积极培育和打造新的消费热点；发挥投资的关键作用，着力推进内涵效益型投资增长；发挥出口的支撑作用，坚持不懈推动开放型经济快速增长。二是必须更加注重发挥改革的红利效应、创新的驱动支撑和市场配置资源的决定性作用，做好发现和培育新增长点的“加法”，做好化解过剩产能和节能减排的“减法”，做好科技、管理、市场、商业模式创新的“乘法”，做好提高劳动生产率和资本回报率的“除法”，通过“加减乘除”四则运算，加快经济结构调整和转型升级步伐。三是必须更加注重发挥自身优势，创造性地对接好、落实好上级一系列改革发展政策和区域发展战略，找准目标定位，做好结合文章，主动借势发展。四是必须更加注重各业协调并进，在巩固提升农业的基础上，重点抓好旅游业、商贸物流业、先进制造业和城镇化，实施“四轮”驱动，进而带动金融、会展、总部经济、养生养老、教育医疗等“多业”快速发展。

　　各位代表，每一次经济大调整，都是资源重新配置、区域竞争最激烈的时期。机不可失，时不我待。我们要进一步强化事业心和责任心，增强紧迫感和使命感，始终做到永不保守、永不满足、永不停顿、永不懈怠，以更扎实的工作，瞄准更高标杆，奋力开创泰安科学发展新局面！

　　三、全面深化改革

　　破除体制机制障碍，激发经济发展活力，关键靠全面深化改革。我们要按照中央要求，结合泰安实际，积极稳妥地把各项改革举措落实到位、取得实效。

　　一是进一步简政放权。深化行政审批制度改革，建立行政审批事项目录清单、行政权力清单、政府责任清单和市场准入负面清单“四张清单”，健全完善政务服务、公共资源交易、便民服务“三个平台”。继续取消和下放行政审批事项，加强事中事后监管。加大政府购买公共服务力度，鼓励社会力量积极参与到更广泛的服务领域。坚持“宽进严管”，进一步深化商事制度改革。

　　二是深化农村改革。全面完成农村土地承包经营权确权登记颁证工作，探索开展农户土地经营权抵押、担保贷款试点。抓好农村集体建设用地使用权和宅基地使用权确权登记颁证工作。强化农村集体“三资”管理，实施以清产核资、资产量化、股权管理为主要内容的集体资产股份合作制改革。规范建设县级农村综合性产权交易平台，并逐步向乡镇延伸、向农村覆盖。进一步深化供销社改革。继续推进集体林权配套改革和国有林场改革，抓好新泰市全国集体林业综合改革试验示范区建设。深化水利投融资体制和水行政管理体制改革，落实最严格水资源管理制度。

　　三是深化财税金融改革。认真执行新《预算法》，健全政府“全口径”预算体系，改进年度预算控制方式，全面推进预决算公开。加强预算绩效管理，推行第三方独立评价。积极推动“营改增”试点扩围，全面清理财税优惠政策。深化财政支出管理改革，大力压减“三公”经费等一般性支出，实施公务用车制度改革。整合规范财政专项资金，逐步取消竞争性领域专项，更多地采取基金引导、政府与社会资本合作等市场化运作模式，扶持经济转型升级发展。改革机关事业单位工资制度，调整工资标准，优化薪酬结构，在县以下机关全面实行职务与职级并行制度，进一步提高基层公务人员工作补贴。积极推进机关事业单位养老保险制度改革。加强政府债务管理，建立借、用、还相统一的地方性债务管理机制。

　　加快金融改革发展，扩大社会融资总量，加大对实体经济，特别是对转方式、调结构的信贷投放和直接融资。大力发展多元股本投资，鼓励各类机构发起设立产业投资基金和股权投资基金。规范发展民间融资，完善多元投融资机制，加快小额贷款公司、融资性担保公司、民间资本管理公司、民间借贷登记服务公司、“金融超市”等普惠金融组织发展。加大农村信用社银行化改革力度，稳妥做好农村合作金融改革试点。推进企业股份制改造，加快企业上市、挂牌步伐。科学规划金融业发展布局，建设金融商务区。加快地方金融信用和信息服务平台建设，优化金融生态环境，防控地方金融风险。

　　四是深化国资国企改革。完善以管资本为主的国资监管体制，推动国有资本合理流动、有效配置，提升国有资产运营效益。加快国有资产资本化步伐，鼓励民营经济、社会资本参与国有企业改制重组，打造一批体制新、机制活、竞争优势明显的混合所有制企业。深化市属国有企业改革，健全公司法人治理结构，加快建立现代企业制度。建立企业负责人经营业绩考核和责任追究制度，规范市属企业负责人职务消费。

　　五是加快事业单位分类改革。抓好事业单位分类工作，基本完成经营类事业单位转企改制任务，研究制定承担行政职能事业单位改革方案。探索推进事业单位管办分离，完善法人治理结构，逐步取消学校、科研院所、医院等单位的行政级别。规范中介机构，基本实现行业协会商会与行政机关脱钩。将社会力量举办的非营利性学校、医院、养老等机构逐步纳入事业法人登记管理。

　　四、大力推进产业结构调整转型

　　加快产业结构调整转型，关乎泰安的长远发展和可持续发展。我们要把经济工作的着力点放在转方式、调结构上，以转求进、以调促优，努力做到调速不减势、量增质更优。

　　（一）坚定不移推进工业经济升级壮大。科学编制全市工业发展布局规划，继续大力实施工业经济转型升级万亿三年行动计划和百亿企业培植计划。加快传统产业尤其是重化工产业转型升级，逐个行业制定转型升级方案，提高终端产品和高附加值产品比重。把发展壮大新材料、新能源与节能环保、电子信息、生物制药和生物育种等战略性新兴产业，作为工业转型升级的着力点，制定发展促进计划，尽快把一些新兴产业培育成主导产业。大力发展高端装备业，围绕做强优势产业和骨干企业，加快工业化和信息化深度融合，推动企业生产过程智能化、生产装备数字化和能源利用效率最大化。加大科技型中小企业培植力度，壮大高新技术产业规模，今年高新技术产业产值占规模以上工业总产值比重提高1.3个百分点以上。加快工业重大项目建设，重点抓好投资80亿元的中国五矿甬泰（泰山）新材料科技园、投资60亿元的正大焦化180万吨冶金焦煤化工、投资60亿元的中美泰山绿色环保产业园、投资30亿元的华新金谷科技园、投资30亿元的泰山云谷互联网产业园等一批转型升级项目。着力提升企业组织化程度，横向依托县市区工业联盟形成企业团队，解决抱团发展问题；纵向依托行业协会形成企业列队，解决产业链条延伸问题。加强企业家队伍和企业管理团队建设，创新企业运营管理模式，向管理要质量、要效益。提高园区集约发展能力，支持泰安高新区等园区创新体制机制，推动园区“空间换地”、“腾笼换鸟”，率先转型升级。

　　（二）在更高水平上推动服务业提档发展。年内服务业增加值占比提高1个百分点以上，服务业税收占比进一步提高。强化旅游业龙头作用，突出内涵提升和品牌打造，加快建设国际著名旅游目的地城市。编制旅游业发展总体规划，健全完善市级旅游业发展管理体制。理顺旅游经济开发区、徂徕山汶河景区管理体制，加快两区开发建设。深度做好旅游和文化融合文章，提升已运营旅游项目品质，同时争取再建成一批质量效益高、可持续发展的精品文化旅游项目。加大旅游宣传促销力度，完善“三日游”工作机制，提高旅游项目整体效益和运营水平。大力发展乡村旅游，建设一批特色鲜明的乡村旅游示范点和集聚区，打造“泰山人家”、“水浒人家”品牌。继续强化旅游综合执法、旅游市场管理、旅游设施建设和旅游服务标准化创建，进一步提高泰安旅游满意度。

　　现代商贸物流业是经济发展新增长点和转型升级新着力点。我们要把发展现代商贸物流业摆上重要位置，借助后发优势，努力实现弯道超越。高起点编制现代商贸物流业发展规划，重点扶持壮大交运集团、岳华能源物流、容大医药等一批物流企业，加快建设新泰天宝现代物流园、润恒冷链物流园、宁阳康平纳四海物流园、东平现代国际物流园等一批物流园区。支持电子商务与快递业协同发展，规划建设电子商务产业园和快递产业园。完善会展经济发展体制机制，对接引进高端国内外会展资源，办好自办展会，打响泰安特色会展品牌。积极发展连锁经营、物流配送，加快城市特色商业街（区）建设，推动传统商贸流通业转型升级。

　　大力发展文化产业，推动文化与农业、工业、商贸流通、电子信息、体育等产业深度跨界融合，支持具有浓郁地方特色和较强影响力的文化企业做大做强，打造一批泰山文化产品品牌、活动品牌和企业品牌，构筑泰安文化产业发展高地。加快发展金融保险、研发设计、信息技术、动漫创意、教育科技、健康养生、家政服务等新兴服务业，满足个性化、多样化消费需求，培育新的消费热点。努力促进大众消费，通过增加收入、完善政策、改善环境，让群众能消费、愿消费、敢消费，使大众消费“汇小溪成江河”。

　　（三）下大气力推动农业发展方式转变。扎实开展千亿斤粮食产能建设和粮食高产创建活动，确保粮食播种面积和产量稳定。深入实施十四大特色优势产业发展规划，抓好国家级、省级“一村一品”示范村镇建设，大力发展优质高效特色农业，新增茶园4000亩、苗木花卉1.5万亩；推进螭霖鱼、四大名药产业化开发。引导农户承包土地经营权规范有序流转，积极培育联户经营、专业大户、家庭农场、农民合作社和农业社会服务组织等新型经营主体，新发展农民合作社200家、家庭农场100家。引导更多的工商资本投向农业，加快推进一产特色化、二产化、三产化和信息化“一产四化”进程，培育壮大一批带动能力强、产品附加值高的产业化龙头企业，新增规模以上农业龙头企业50家。加大农机推广力度，提高农业机械化水平。完善基层农业技术推广体系和运行机制，开展科技进村入户活动，打通农业先进技术转化应用“最后一公里”。加快实施5个小农水重点县工程，构建“旱能浇、涝能排”农田水利工程体系。加强防洪工程和雨洪资源利用工程建设。继续实施农村公路网化工程，新建改建农村公路500公里。建立农产品质量安全监管体系，整建制推进农产品质量安全县建设。认真抓好动植物疫病防控、新型职业农民培训、农村饮水安全、强农惠农富农政策落实等工作。

　　各位代表，推进产业结构调整转型，是一项长期任务，不可能毕其功于一役，我们要在以下几个方面长期坚持、狠下功夫。

　　一是以更大的力度抓好项目建设和招商引资。投资仍是拉动经济增长最直接和最有效的手段。要切实把握好“投向、投量、投速、投效”的关系，持之以恒把项目建设和招商引资作为推进转型升级、培育新增长点的重要举措。要进一步完善指挥部工作机制，落实领导干部包保重点项目、检查考核、跟踪服务等制度，争取再开工投产一批税收贡献大、带动能力强、科技含量高的大项目、好项目，使经济发展更有质量、更具活力、更可持续。年内重点抓好120个左右市级重点建设项目，加快实施100个左右前期推进项目，策划储备100个左右能够拉长产业链的重大项目。同时，积极做好济泰东部高速公路、济泰城际铁路、聊泰铁路、泰安机场等重大基础设施项目前期工作。创新招商理念和方式，改变拼资源、拼价格、拼土地的传统模式，更多地依靠环境、人才和服务，招大引强、招新引优、招才引智，努力引进一批引领型、补链型、核心型产业项目，助推产业转型升级。更加注重项目质量和效益，严格项目联合审查，坚决制止盲目投资和高耗能、高污染、低水平重复建设。

　　二是以更大的力度提升对外开放水平。深入实施开放兴市战略，重视加强开放型人才队伍建设，潜心打造国际化营商环境，坚持引资引技引智相结合，加快构建以开放型经济为核心的全方位对外开放体系。进一步扩大利用外资规模，大力引进世界500强和跨国知名企业，同时鼓励现有外资企业通过兼并、收购、控股、参股等形式做大做强，引导外资参与产业转型升级和区域协调发展。多措并举稳定外贸，启动出口农产品质量安全示范市创建工作，抓好科技兴贸出口创新基地、外贸转型升级基地和公共服务平台建设；鼓励企业扩大能源原材料、关键技术、关键设备及零部件进口，带动产业转型升级。支持有条件的企业向全球布局产业链，搞好境外资源合作开发和优势产能转移，推进境外合作园区建设，开拓高端劳务市场。大力发展服务贸易和服务外包。加快建设泰安高新区海关综合监管区和青岛保税港区泰安（新泰）功能区。积极融入省“一圈一带”、国家中原经济区、“蓝黄”两区和长三角、京津冀等区域发展战略。

　　三是以更大的力度加强生态文明建设。强化生态环境倒逼作用，确保完成“十二五”节能减排和化解过剩产能硬任务。积极发展循环经济，抓好全国资源综合利用“双百工程”示范基地和新泰全国循环经济示范创建市、国家资源枯竭型城市转型发展等工作。认真落实大气污染防治“蓝天工程”三年行动计划，下大气力治理重点行业、重点区域大气污染尤其是扬尘污染，稳妥推进主城区重污染企业搬迁或转产。加强水生态文明建设，扎实开展全国水生态文明城市试点。全面加快汶河、康王河国家湿地公园建设，抓好天泽湖、柴汶河等人工湿地水质净化项目。大力实施造林绿化，完成成片造林20万亩，创建国家森林城市争取通过国家验收。推进节约集约用地，提高各类土地使用效率。健全生态文明制度体系，深入开展生态文明乡村行动，全面推动生态文明示范区建设，构筑泰安生态文明建设高地。

　　四是以更大的力度促进创新创业和创造。深入实施创新驱动战略。充分发挥企业创新主体作用、科研院所和大专院校要素支撑作用、泰山科学院平台载体作用，健全完善政、产、学、研、金有机结合协同创新机制，加快建设一批高水平科技创新和服务平台。加大对企业科技研发、技术创新扶持力度，以实施重大科技项目为支撑，支持优势产业和骨干企业承担国家、省重大专项和重大计划，力争在云计算、大数据、新材料、新工艺等方面攻克一批关键技术。

　　大力实施创业富民工程。认真落实中央、省加快民营经济发展一系列政策措施，放宽准入领域，支持民间资本进入基础产业、基础设施和金融、教育、文化、养老、医疗、体育等领域。健全完善中小微企业公共服务平台，加快中小微企业创业辅导基地、创业园区等载体建设，激发和释放民营市场主体活力。用好中小微企业发展专项资金，大力实施个升微、微升小、小升中、中升大“四升”计划和民营经济倍增计划。强化创业观念引导，加大政策扶持力度，完善大学生创业“三级孵化”模式，建设农民工返乡创业园。

　　积极实施人才强市战略。健全完善人才培养引进和使用激励机制，依托重大项目、重点企业、科技园区、创新平台和特色优势产业，加大对高层次创新人才、产业领军人才和高技能人才的培养引进力度，构筑泰安人才聚集高地。

　　我们相信，只要全市营造形成了大众创业、万众创新、人才创造的良好环境和浓厚氛围，我市产业转型升级和跨越发展就指日可待！

　　五、加快新型城镇化建设

　　推进以人为核心的新型城镇化，建设好我们共同生活的家园，是每个人的希望和梦想，需要人人参与，共建、共爱和共享。

　　一是更加注重规划的引领作用。坚持”先规划、后建设”，加快编制全市城镇化发展规划，推动城乡规划与产业发展规划、土地利用规划、环境保护规划等“多规”融合。加快泰城总体规划和历史文化名城保护规划修编，优化空间布局，打造城市特色，拓展发展空间。加强泰城重点片区概念性规划和城区单元控制性规划编制，完善公共服务设施、城市风貌、城市绿道、城市交通等专项规划。抓好小城镇总体规划编制研究，规划发展好农村新型社区。坚持严管重罚，强化批后监管，坚决遏制各类违法违规建设行为。

　　二是更加注重城镇功能配套完善。泰城重点加快推进5大城市综合体和10大城市片区建设，其中高铁新区年内完成投资100亿元，财源街中央商务区、历史文化轴片区要有突破性进展。积极探索PPP等城建项目投融资模式，加大城市基础设施建设投入。泰城继续实施104国道改建、万官大街、灵山大街、高铁出站口改造等建设工程，完成温泉路南段、东岳大街拓宽、傲徕峰路、紫欣街等建设工程。加快城市供水配水管网改造、供热管网铺设和“汽改水”，推进供水、供热等基础设施向镇村延伸。加强城市雨污分流管网改造和排水防涝设施建设。高度重视城市饮水安全，完成环湖水厂和小安门水库引水建设工程，抓好“角峪-彩山-大汶河-旧县水源地联合供水工程”前期准备工作。改造升级第一、二污水处理厂，建设完成第四污水处理厂，加快完善城区污水管网。加大城乡环境综合整治力度，重点抓好泰城南部、城乡结合部、镇村环境卫生整治，泮河、开元河等河道治理和城区农贸市场建设。

　　三是更加注重城市精细化管理。深化城市管理体制改革，进一步完善集管理、执法、服务于一体的“大城管”体制。强化两区和办事处城市管理职能，实行泰城分级和数字化、网格化管理，年底力争实现泰城核心区域数字化管理全覆盖。抓好小区物业管理，提高物业管理服务水平。加强城市文明和社会公德教育，创建全国文明城市，引导市民像爱护自己的眼睛一样爱护我们的城市。

　　四是更加注重城乡一体化体制建设。深化户籍制度改革，促进有能力在城镇稳定就业和生活的常住人口有序实现市民化。推进城镇基本公共服务常住人口全覆盖，重点放在教育、就业、养老、医疗卫生、住房保障、城乡环卫、邮政普遍服务等民生领域。大力发展公共交通，推动城乡公交资源衔接融合、有序发展。探索将农民工纳入住房公积金制度，扩大制度受益面。

　　六、加强民生保障和社会建设

　　民之所盼，政之所往；民之所需，政之所向。让群众生活得更好、更有尊严，是各级政府义不容辞的责任。新常态下，应更加注重保障基本民生，加强社会建设。

　　一是健全就业和社会保障体系。实施更加积极的就业政策，继续抓好高校毕业生、农村转移劳动力、城镇困难人员、退役军人和“零就业”家庭人员就业，落实好农民工技能提升、权益保障和公共服务3个三年行动计划。坚持“全覆盖、保基本、多层次、可持续”，健全社会保障制度，扩大社会保险覆盖面；完善落实城乡居民基本医疗保险、大病保险和临时救助制度；进一步提高城乡低保、五保供养和居民基本医疗保险财政补助标准。加快养老服务设施建设，新增养老床位3000张。抓好0-6岁残疾儿童康复救助工作和残疾人康复中心、托养中心建设。积极开展精准扶贫，加快实施东平湖移民避险解困工程。开工建设保障房3.5万套，有序推进公租房和廉租房并轨，探索把符合条件的企业建房纳入保障房管理。

　　二是繁荣发展各项社会事业。坚持教育优先发展，推进基础教育综合改革，完善学业水平考试和综合素质评价办法，探索建立现代学校制度；实施第二期学前教育三年行动计划；理顺义务教育学制，全面改善薄弱学校办学条件，落实困难救助政策，提高义务教育均衡化水平；做好省级区域信息化试点工作，加快实现“校校通、班班通、人人通”，扩大优质教育覆盖面；完善现代职业教育体系，加强校企合作，推进职业特色名校建设。加强对泰山文化资源的挖掘和阐发，创作一批富有时代精神和泰安特色的文艺精品，弘扬中华优秀传统文化；完善现代公共文化服务体系，加强基础设施建设，大力实施文化惠民工程；积极开展全民阅读活动，推进公共博物馆、纪念馆、爱国主义教育基地和文化馆、图书馆、美术馆免费开放；实施“乡村记忆工程”，加大对古村、古街、古巷、古建筑保护利用力度。深化医药卫生体制改革，重点推进县级公立医院综合改革，探索建立分级诊疗制度；巩固国家基本药物制度实施成果，提高基本公共卫生服务补助标准，做好卫生应急和重大疾病防控工作。积极发展中医药事业。抓好市中心医院高新区院区等医疗卫生设施建设。坚持计划生育基本国策不动摇，全面推进转型发展，实现免费孕前优生检查城乡全覆盖，争创全国计划生育基层群众自治示范市。大力发展体育事业和体育产业。依法管理宗教事务，增进民族团结。认真抓好国防动员、人民防空、民兵预备役、双拥共建和优抚安置工作。繁荣发展其他社会事业。

　　三是加强和创新社会治理。深化社会组织登记管理体制改革，积极培育和发展基层社会组织。探索建立社区、社工、社会组织、社区志愿者“四社”联动机制，推动社区服务专业化、社会化。深入推进平安泰安建设，完善立体化社会治安防控体系，严厉打击各类违法犯罪活动，提升群众安全感。建立完善基层网格化、社会化综合服务平台，及时回应群众诉求。推进涉法涉诉信访改革，把信访纳入法治轨道。加强矛盾纠纷排查化解，重视解决好土地征用、房屋拆迁、企业改制、环境污染治理等引发的矛盾和问题。更加重视食品药品和安全生产监管，做好应急管理、防灾减灾、抢险救援等工作，确保社会和谐安定。

　　七、加强政府自身建设

　　打铁还需自身硬。经济新常态、发展新任务、群众新期待，要求各级政府必须在以下几个方面加强建设、改进提高。

　　一是强化依法行政，建设法治政府。政府的一切权力来自人民、源自法授。全体政府工作人员要强化法律权威意识，坚持依法办事，始终做到学法、尊法、守法、用法。坚持依法科学决策，严格行政决策程序，完善政府法律顾问制度，建立重大决策终身责任追究制度和责任倒查机制。坚持严格规范、公正文明执法，落实重大执法决定法制审核、备案制度和执法案件评查、评议考核制度，健全完善行政执法争议协调机制和纠错问责机制。坚持依法向人大报告工作和向政协通报工作，认真落实人大及其常委会的决议、决定，自觉接受人大的法律监督和政协的民主监督，办理好人大代表议案、建议和政协提案。

　　二是强化工作创新，建设效能政府。如何做好经济发展新常态下的政府工作，对我们来说是一个新课题。全体政府工作人员要不断加强对新知识的学习和研究，在素质本领上适应新常态，不说外行话、不干外行事、不当外行人。坚决冲破陈旧观念和思维定势，积极探索新形势下做好政府工作的新思路、新办法，勇于创新、勇于实践。始终把干事创业谋发展作为最大责任，坚决克服等待观望和畏难发愁情绪，坚决纠正懒政怠政、“为官不为”行为，切实做到主动作为、善做善成。扎实做好党的群众路线教育实践活动后续整改、成果拓展工作，严格落实中央八项规定，加大“庸懒散”等突出问题整治力度。落实行政问责、效能监察、举报投诉制度，深入开展群众满意度调查、机关作风和政风行风评议，提升政务服务效率和水平，构筑泰安营商环境高地。

　　三是强化为民宗旨，建设务实政府。评价政府工作，关键是看干成了多少事，能否让群众得到更多实惠。各级政府要始终秉持求真务实工作作风，不搞花拳绣腿，不搞形象工程，不搞短期行为。认真搞好调查研究，特别要高度关注深化改革、转型升级、创新驱动、社会治理等重大问题，增强工作的前瞻性和主动性。进一步增强群众观念和为民情怀，广泛深入基层、深入群众，多到困难多、矛盾多的地方去，真情纾民困，真心排民忧，真实解民难。进一步强化目标任务责任制，层层落实责任，层层传导压力，加强督查考核，切实把政府各项重点工作、为民要办的10件民生实事落到实处。

　　四是强化监督制约，建设清廉政府。严格落实党风廉政建设责任制，严格遵守党的纪律和廉洁自律各项规定，做到思想上警钟长鸣，行为上自觉自律，守法纪、讲规矩。全面推进政务公开，切实加强制度建设，做到既让权力在阳光下运行，又把权力关进制度的笼子。加强行政监督、司法监督、审计监督、社会监督和舆论监督，加大违法违纪案件查办力度，形成正风肃纪的严厉高压态势。全体政府工作人员都要珍惜组织和人民赋予的权力与岗位，清清白白做人，干干净净干事，树立政府良好形象。

　　各位代表，“长风破浪会有时，直挂云帆济沧海”。让我们紧密团结在以习近平同志为总书记的党中央周围，在中共泰安市委的坚强领导下，不畏艰难，开拓创新，埋头苦干，全面完成“十二五”规划目标任务，为加快推进富民强市、建设幸福泰安进程而不懈奋斗！
